# Effects of MicroRNAs on Fucosyltransferase 8 (FUT8) Expression in Hepatocarcinoma Cells

**DOI:** 10.1371/journal.pone.0076540

**Published:** 2013-10-09

**Authors:** Cinzia Bernardi, Ugo Soffientini, Francesco Piacente, Michela G. Tonetti

**Affiliations:** 1 Department of Experimental Medicine and Center of Excellence for Biomedical Research, University of Genova, Genova, Italy; University of Insubria, Italy

## Abstract

Fucosyltransferase 8 (FUT8) catalyzes the transfer of α1,6-linked fucose to the first N-acetylglucosamine in N-linked glycans (core fucosylation). Increased core fucosylation has been reported during hepatocarcinogenesis, in both cell-associated and secreted proteins. Accordingly, increased core fucosylation of α-fetoprotein and α1-antitrypsin is currently used as a diagnostic and prognostic indicator. The present study provides new evidences that FUT8 can be regulated also through miRNA-mediated mechanisms. Using microRNA/target prediction programs, we identified miR-122 and miR-34a seed regions in the 3′ untranslated region (3′UTR) of FUT8. Then we used human and rodents hepatocarcinoma cell lines to evaluate the impact of transfection of miR-122 and miR-34a mimics on FUT8 mRNA and protein levels. This study demonstrated that forced expression of these miRNAs is able to induce a decrease of FUT8 levels and also to affect core fucosylation of secreted proteins. The ability of miR-122 and miR-34a to specifically interact with and regulate the 3′UTR of FUT8 was demonstrated via a luciferase reporter assay. Since miR-122 and miR-34a downregulation is a common feature in spontaneous human hepatocarcinoma, our finding that these miRNAs are able to target FUT8 3′UTR suggests that, together with transcriptional and other post-transcriptional systems, a miRNA-mediated mechanism could also be involved in the increased core fucosylation observed in liver tumors. Moreover, these findings also point out that miRNAs may be widely involved in the regulation of glycosylation machinery.

## Introduction

MicroRNAs (miRNAs) are an abundant class of short endogenous, non coding RNA about 20–25 nucleotides in length. They can pair to a mRNA and thereby induce the post-transcriptional repression of that protein-coding message, either by transcript destabilization, translational repression or both [1]. They are generated from sequential processing of primary miRNA transcripts by Drosha and Dicer. In mammals, mature miRNAs are integrated into a RNA-inducing silencing complex (RISC) and associate with the 3′ untranslated regions (3′ UTR) of the specific target messenger. Computational analyses predict the existence of hundreds of different miRNAs, which are either highly conserved among different species or greatly vary among organisms. It has been postulated that each miRNA may control tens to hundreds of genes and that altogether they can control a great percentage of the human genes and most of the cellular pathways [2].

Increasing evidences indicate that miRNAs play an important role in several physiological and pathological processes, such as cell growth and differentiation, development, cancer and viral infections [1]. In particular, dysregulation of miRNA expression may affect known oncogenes and tumor suppressor genes, thereby having implication in carcinogenesis [3]. In fact, alteration in miRNA expression is considered a hallmark of cell transformation.

It is also well recognized that aberrant glycosylation is a marker of tumoral transformation, affecting cell growth, migration and tumor metastasis [3]. Thus, it is conceivable to predict that, in addition to other already identified mechanisms, miRNAs may play also a role in the aberrant glycosylation observed in cancer cells. Indeed, few reports recently appeared in the literature, which demonstrated the involvement of specific miRNAs in the control of GalNT7, a key enzyme involved in the formation of mucin-type O-linked glycans [4], [5], [6]. However, the role of miRNAs in the control of glycosylation remains mostly unexplored. To extend the information on this issue, we chose fucosyltransferase 8 (FUT8) as a model, in order to establish if miRNAs could be involved in regulating its expression in hepatocarcinoma cells. FUT8 is the only enzyme responsible for α1,6-fucosylation of N-glycans, catalyzing the transfer of fucose from GDP-L-fucose to the asparagine-linked N-acetylglucosamine [7]. Core fucosylation has been demonstrated to be essential for signalling of several growth factors and adhesion molecules, such as EGF, E-cadherins TGFβ, and integrins [8]–[12], thus it can play a fundamental role during carcinogenesis. It has been reported that FUT8 activity is increased in hepatocarcinoma cells compared to the surrounding tissues and that this also results in increased α1,6-fucosylation of α-fetoprotein and α1-antitrypsin [13]. These tumor markers containing higher amounts of core fucose are in fact consistently found in serum of patient with liver cancer already in the early stages of the disease and their presence has been associated to a poor prognosis.

Using several miRNA-target prediction tools, we identified several miRNAs potentially able to interact with FUT8 3′UTR. Among them, miR-122 and miR-34a, were further chosen for an experimental validation, since their dysregulation during hepatocarcinogenesis is well known. miR-122 is the most abundant miRNA in adult hepatocytes, accounting for about 70% of total miRNA content, while it is expressed at low levels during liver development [14]. miR-122 has been also reported to be specifically and consistently downregulated in most spontaneous liver tumours and in almost all hepatocarcinoma cell lines [13]. In hepatocellular carcinoma it was shown to modulate cyclin G and other important targets for proliferation and apoptosis [14]. Systemic administration of LNA- or PNA-based miR-122 antagonists in mice lead to upregulation of a large set of genes in liver, as revealed by genome-wide expression profiling, and FUT8 proved to be among the upregulated genes in the liver after systemic administration of a LNA-antagomir-122, both after acute administration and chronic treatment (3 weeks) of mice [15]. On the other side, *miR-34a i*s a transcriptional target of p53 and it has been reported to be downregulated in many types of cancer, including hepatocarcinoma [16].

Here, we report the effects of transient transfection of miR-122 and miR-34a mimics on expression levels of FUT8 mRNA and protein and on core fucosylation of secreted glycoproteins in human and rodents hepatocarcinoma cell lines. This study demonstrated that ectopic expression of both miR-122 and miR-34a was able to significantly decrease FUT8 levels and also to affect core fucosylation of secreted proteins, suggesting that a miRNA-mediated mechanism could also play a role in the dysregulation of core fucosylation observed in liver tumors.

## Materials and Methods

### Prediction of miRNA Sites in the 3′UTRs

The presence of miRNA recognition sites in the 3′UTR of FUT8 and ALDOA was performed using TargetScan (www.targetscan.org), PicTar (http://pictar.mdc-berlin.de) and Miranda (www.microrna.org) tools [2]. These programs predict biological targets by searching for the presence of conserved 8 mer and 7 mer sites that match the seed region of each miRNA.

### Cell Lines

Cell lines were obtained from ICLC (Interlab Cell Lines Collection, Genova, Italy). Human HepG2 and HeLa were grown in Dulbecco Modified Eagle’s Medium (DMEM), mouse Hepa1C1C7 and rat HTC cell lines were grown in DMEM F12, supplemented with 10% and 5% FCS, respectively. Cells were grown until 70% confluence and then harvested by trypsinization. Cells were routinely tested for mycoplasma contamination.

### Luciferase Assay

The complete 3′UTRs of both human FUT8 and ALDOA were amplified by PCR, using two couples of primers containing SpeI and HindIII restriciton sites. The amplification products were cloned downstream of firefly luciferase in the pMirReport vectors (Ambion) using the above indicated restriction sites. The resulting vectors are indicated as pMir/FUT8 and pMir/ALDOA. HeLa transient co-transfections of pMirReport vectors and miR-122a and miR-34a mimics were made using Attractene (Qiagen), following the manufacturer’s protocol. Transfection was performed in 96-well plates, using six well replicates for each sample. HeLa were plated at 2×10^4^ cells/well in 100 µl of serum containing medium and for each well 100 ng of either pMir/FUT8 or pMir/ALDOA and 1.6 pmols miRNA mimics were added together with 0.75 µl of Attractene in 50 µl of serum-free medium to obtain a final volume of 150 µl/well. Empty pMirReport vector and All Star Negative Control (Qiagen) were used as controls. After transfection, plates were incubated for 24 hours and medium was changed. Luciferase activity was then measured using the One-Glo Luciferase Assay System (Promega) according to manufacturer’s protocol. Reagent was added to the wells and the resulting mixture was quickly transferred in a 96-well white plate (Costar). Light emission was recorded with FluoStar Optima (BMG Labtech). Data were expressed as relative luminescence units (RLU).

### miRNA Mimic Transfection

Transient transfection experiments were performed using miRNA mimics, chemically synthesized double-stranded RNAs which mimic mature endogenous miRNAs once transfected into cells. HepG2 cells (3×10^5^) were seeded in triplicate in each well of 12-well-plates in antibiotic-free medium and transfection was immediately performed using the HiPerFect Transfection Reagent as recommended by the supplier (Qiagen) with 300 ng of miRNA Mimic 34a or 122 (Qiagen), Hepa1C1C7 and HTC cell lines (2×10^5^) were seeded as mentioned above and 150 ng of miRNA Mimic 34a or 122 were used. AllStars Negative Control siRNA was used as a non-silencing control. Cells were further incubated for different times and then they were washed three times with ice cold PBS before RNA and protein extraction. Total RNAs and proteins for each time point and condition were obtained from the same well, using the Trizol procedure (Invitrogen) and following the provided protocol.

Total fucosylation was investigated by lectin blot analysis of secreted proteins in the culture medium. For this purpose, transient transfection was performed in three 35 mm diameter dishes with 10^6^ hepatocarcinoma cells and with 500 ng of miRNA mimics 34a, 122 or negative control. After 24 hours from transfection cells were extensively washed to remove FCS, and serum-free medium (Optimem, Gibco) was added. After further 24 hours, the medium was recovered and concentrated by ultrafiltration, using YM-10 filters (Millipore); proteins were determined by Bradford assay (Biorad).

### RNA Retro-transcription and qRT-PCR

Purified total RNA was resuspended in DEPC-treated water and the quality was determined by 260/280 ratio. For miRNA analysis, 500 ng of RNA were polyadenylated and retro-transcribed using the NCode miRNA First Strand cDNA Synthesis Kit (Invitrogen) according to the manufacturer’s protocol. Retrotranscription of mRNA was performed using the the QuantiTect Reverse Transcripition Kit (Qiagen) as recommended by the supplier. This kit ensures complete removal of genomic DNA; however, samples in which reverse transcriptase was omitted were used as controls to exclude DNA contamination. Efficiency of mimic transfection was determined by qRT PCR, using the sequences of mature miR-122 or miR-34a as forward primers, and a primer supplied by the Ncode miRNA First Strand cDNA Synthesis Kit (Invitrogen) as an universal reverse primer. Small nuclear RNA U6 was used as internal control. Target mRNAs were analyzed by qRT-PCR using the following primers: FUT8 For, 5′-CCCGTCCTCCATATTTACCCTTG; FUT8 Rev, 5′-ACTGAGACACCCACCACACTG; ALDOA For, 5′-CACCGAGAACACCGAGGAGAAC; ALDOA Rev, 5′-CCGCCCTTGGATTTGATAACTTGG.

Preliminary experiments were also performed to determine the most reliable housekeeping gene to use for normalization. Glyceraldehyde-3P dehydrogenase and β-actin were then chosen as internal controls. PCR was performed with the iQ5 Multicolor RealTime PCR Detection System and the 2x iQ SYBR Green Supermix (Bio-Rad), using 96-well plates (Axygen). A two-step program with annealing at 60°C was used for all samples. Melting curve analyses were used to ensure specifity.

### Western and Lectin Blot Analysis

Proteins, 10 µg for each sample, were run on a 10% SDS-PAGE and then transferred overnight to PVDF membrane. Membrane was blocked with 5% non fat dry milk in PBS containing 0.1% Tween20 for one hour and then probed with anti-ALDOA rabbit polyclonal antibody (Sigma) or anti-FUT8 monoclonal antibody (kindly provided by Prof. Naoyuki Taniguchi, RIKEN, Tokyo). Detection was achieved using a secondary anti-rabbit or anti-mouse HRP-conjugated IgG (Pierce) using a long lasting ECL kit (EuroClone). After detection, membranes were reprobed using an anti-β-actin polyclonal antibody (from Clontech) to confirm equal protein loading in each well.

Lectin blot was performed as previously reported [17]. The membrane was blocked using 3% BSA in TBS/Tween. Biotin-labeled LCA lectin (Vector Laboratories) followed by streptavidin-HRP conjugate (Millipore) were used for detection. Parallel samples (15 µg) were also treated with protein N-glycanase F (PNGase F) (Roche) to remove N-linked glycans and confirm the specificity of lectin detection using the published procedure [14]. Equal protein loading and blotting in the different lanes was determined by staining of the PVDF membrane by Ponceau S.

### Statistical Analysis

Data are presented as means ± SD. Statistical analyses were done by Student’s t-test using the GraphPad 5 software (Prism).

## Results

The tools used for analysis predicted the presence of a 8 mer site matching the seed region of miR-34a and a 7 mer-1A seed region for miR-122 in the human, mouse and rat 3′UTRs. Since ALDOA 3′UTR also contains the sites for potential recognition by both miRNAs and it has been already experimentally validated to be a target for miR-122, we decided to use the human enzyme as a positive control for our experimental protocols.

**Figure 1:**
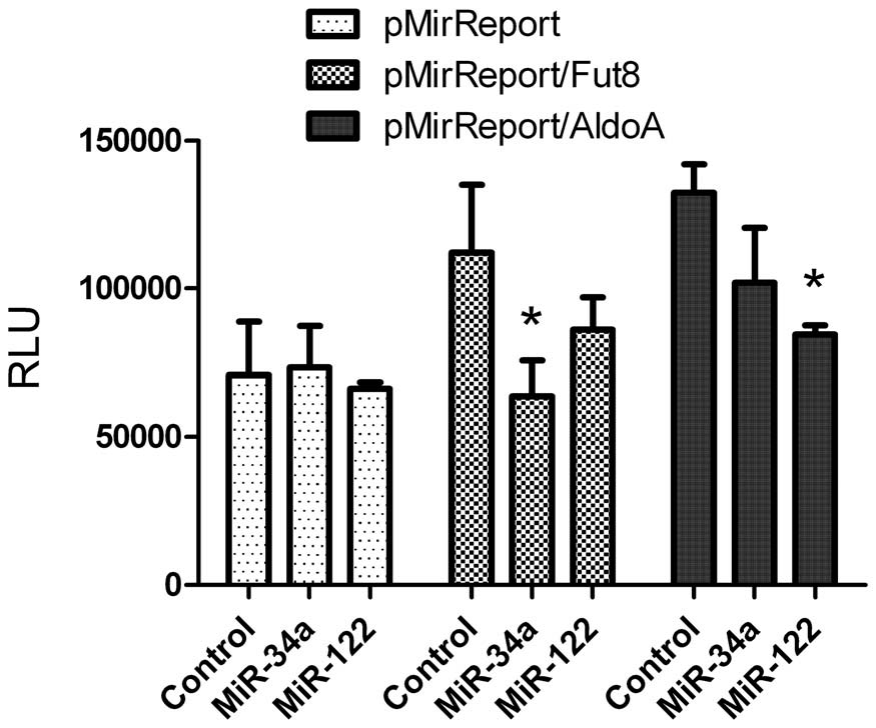
Effects of miR-122 and miR-34a on FUT8 and ALDOA 3′UTRs in a luciferase reporter assay. For each experiment, HeLa cells were seeded in 96 plates (six replicates for each condition) and they were then co-transfected with either miR-122 or miR-34a mimics and with empty pMiR-Report, with pMiR-Report containing downstream FUT8 3′UTR (pMiR-Report/FUT8) or ALDOA 3′UTR (pMiR-Report/ALDOA). Luciferase activity was determined after 24 hours post-transfection. Control = AllStar siRNA negative control. Data were expressed as luminescence units (RLU) means ± SD of six replicates in three independent experiments. *p<0.05.

As a first step, we analyzed if miRNA mimics were able to specifically interact with the 3′UTR regions of our target proteins. For this purpose, we cloned the complete human 3′UTRs downstream of a luciferase reporter gene and we then analyzed luciferase activity after co-transfection of the pMir-Report constructs with miR-122 and miR-34a mimics in HeLa cells. As shown in Figure 1, the presence of the 3′UTR region of both FUT8 and ALDOA “per se” was able to increase luciferase activity compared to the empty vector, possibly by stabilization of the transcripts. However, co-transfection of miR-122 and miR-34a mimics decreased luciferase activity for both target genes, indicating that the miRNAs were able to interact specifically with the 3′UTRs. The effects of miR-122 observed for ALDOA 3′UTR were comparable to those already reported using a similar reporter system [15].

**Figure 2:**
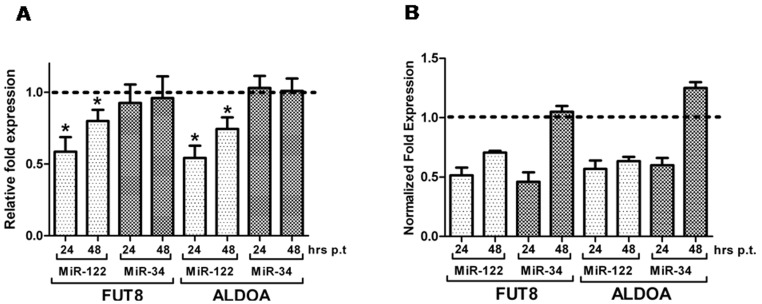
Effects of miRNA transfection HepG2 cells. Cells were transfected with either the AllStar siRNA negative control, miR-122 mimic or miR-34a mimic in 12 well plates. After 24 and 48 hours total RNA and proteins were extracted. (A) mRNA levels were determined by qRT-PCR. Data were reported as relative fold expression compared to controls (indicated by the dotted line) and they were means ± SD of seven independent experiments. (B) Total proteins of HepG2 were recovered after 24 and 48 hours post-transfection of AllStar siRNA negative control, miR-122 or miR-34a mimics. After SDS-PAGE separation and blotting, the membranes were probed sequentially using antibodies against FUT8, ALDOA and β-Actin. Band intensities were measured by densitometry and the values obtained for FUT8 and ALDOA were normalized using β-Actin. Data are expressed as normalized levels relative to the cells transfected with the negative control (indicated by the dotted line). Data were obtained from three independent experiments. *p<0.05.

The direct effects of miRNAs on FUT8 expression were determined after transfection of miR-122 and miR-34a mimics in the hepatocarcinoma cell lines. Transfection efficiency of the mimics was initially evaluated by qRT-PCR and we observed a high increase of the levels of intracellular miRNAs at 24 hours post-transfection, already declining at 48 hours (not shown). FUT8 and ALDOA mRNA expression in human HepG2 cells was then analyzed by qRT-PCR. We initially tested different housekeeping genes, in order to verify that either transfection procedure or mimics did not interfere with their expression. Glyceraldehyde-3P dehydrogenase (GA3PDH) and β-actin were chosen as reference genes. Figure 2 reports the effects of miR-122 and miR-34a mimics on mRNA and proteins expression for FUT8 and ALDOA at 24 and 48 hours post-transfection. Mimic miR-122 was able to induce a decrease of mRNA levels for both genes, which was maximal at 24 hour post-transfection (Figure 2A). This finding is in agreement with the previous reported data which indicated that the systemic administration of antagonists of miR-122 was able to affect also mRNA levels in the mouse liver for both Fut8 and AldoA [*14*]. However, we were not able to demonstrate any significant effect of miR-34a on messenger levels, suggesting that only a translational repression may occur. Indeed, proteomic studies have shown that after transient transfection of miR-34a in HepG2 cells, only few of the of validated miR-34a targets showed a good correlation between mRNA and protein levels [16]. Protein expression was determined using Western blot (Figure 2B); results are reported as relative densitometric levels of expression of FUT8 and ALDOA obtained from three independent transfections. Mimics miR-122 and miR-34a were both able to decrease FUT8 and ALDOA protein levels. However, while miR-122 showed a prolonged effect also at 48 hours, miR-34a induced only a transient inhibition, which was completely reversed at 48 hours.

**Figure 3:**
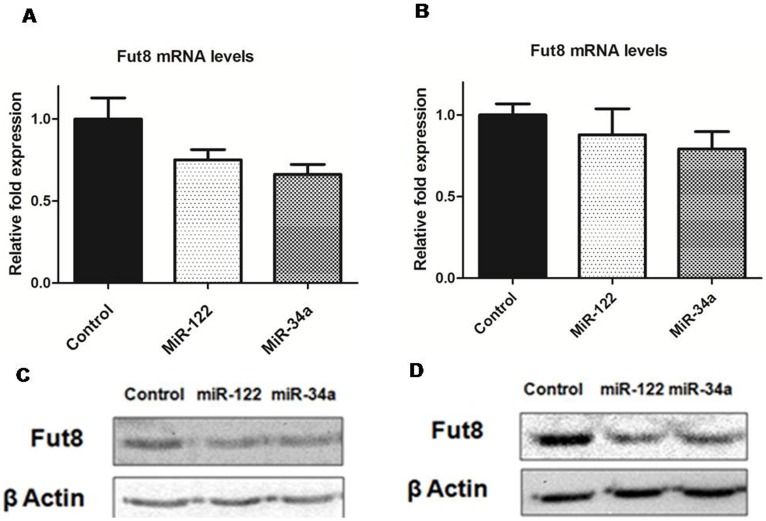
Effects of miRNA transfection on Fut8 protein expression in rodent hepatocarcinoma cell lines. mRNA levels were determined by real-time PCR and were normalized to the value obtained for cell transfected with the siRNA negative control. Western blots were performed using anti-FUT8 antibody; after stripping the same membrane was probed with an anti-β-actin antibody. Data are from one representative experiment for each cell line. Mouse Hepa1C1C7 cell line: Fut8 mRNA (A) and protein (C) levels. Rat HTC cell line: Fut8 mRNA (B) and protein (D) levels.

To further confirm the effects of the two miRNAs on FUT8 also in other experimental models, we analyzed the effects of miR-122 and miR-34a transfection on mouse Hepa1C1C7 and rat HTC hepatocarcinoma cell lines. Figure 3 reports the data obtained from one representative experiment for each cell line. Real-time PCR revealed again a slight decrease of Fut8 mRNA levels, which were more pronounced at 24 hours post-transfection. However, in both rodents cells lines we observed an effect on Fut8 mRNA induced also by miR-34a (Figure 3A and 3C). Western blot analyses revealed that both miR-122 and miR-34a are able to induce also a decrease in Fut8 protein levels (Figure 3B and 3D), confirming the data already observed for human HepG2 cells.

**Figure 4:**
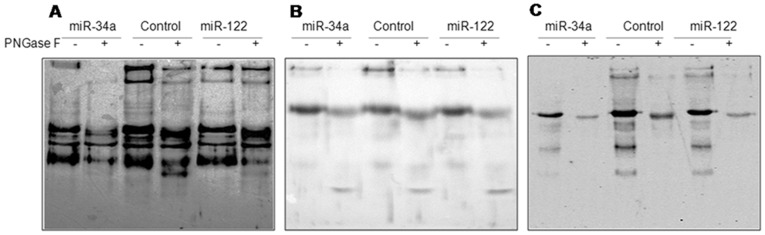
Lectin blot analysis of culture media from HepG2, Hepa1C1C7 and HTC cells after miRNA mimic transfection. Secreted proteins recovered from cell culture supernatants were run on a 10% SDS-PAGE gel and proteins were then blotted on a PVDF membrane. The membrane was probed with biotinylated-LCA and revealed with streptavidin-HRP. Parallel samples were also extensively treated with PNGase F in order to remove N-linked glycans and ensure the specificity of the signal. Representative experiments are shown.

Finally, we analyzed the levels of core fucosylation on global secreted proteins using *Lens culinaris agglutinin* (LCA) lectin blot. LCA recognizes sequences containing α-linked mannose residues; however, the presence of α-1,6 linked fucose residue attached to the N-acetylchitobiose portion of the core oligosaccharide markedly enhances affinity. A slight decrease in signal intensity in several bands was consistently observed by densitometry, in particular for some high molecular weight proteins from miRNAs transfected cells (Figure 4), suggesting reduced levels of core fucosylation. Treatment with PNGase F was able to reduce intensity in most of the bands of interest, confirming the presence of N-linked glycans in these proteins. Staining with Coomassie blue of a gel run in parallel and with Ponceau S of the PVDF membrane confirmed equal protein loading and transfer in each lane.

## Discussion

Over the past few years, miRNAs have emerged as key components in the control of cell functions. They have been recognized to play a fundamental role in controlling protein expression at a post-transcriptional level. miRNAs activities have been categorized depending on the effects on target expression. In fact, interactions of miRNAs with their targets can induce a complete repression of the expression (switch targets); alternatively miRNAs can have an auxiliary activity able to modulate protein expression, which is mainly controlled by other transcriptional or post-transcriptional mechanisms (tuning targets) [1]. In these ways, the miRNAs add a further level of gene control that integrates with transcriptional and other regulatory processes to expand the complexity of metazoan gene expression. Moreover, microarray expression profiling, and recently also proteomic studies, provided evidences that single miRNAs can control large sets of genes. Similarly, single genes can be the target of several miRNAs, acting either in a synergy or in different tissues or at different times during development. Thus, microRNAs suit very well the need to coordinate and finely tune the expression of different proteins that are involved in the same pathway or function. Since miRNAs can act not only inducing mRNA degradation, but as translational repressors, they can also function as reversible regulators able to quickly release sequestered messengers. This indicates an active cross-talk between miRNAs and other RNA binding proteins, affecting the final gene expression.

For all these reasons, the machinery for glycoconjugates formation represents an attractive target for miRNA-mediated control mechanisms, in addition to the several already identified systems. A first evidence that a miRNA can play a role in regulating the expression of enzymes involved in protein glycosylation was obtained serendipitously in a study aimed to analyze the effects of miR-378 on nephronectin, a ligand for integrin α8β1 [4]. In fact, a competition for miR-378 binding was observed between nephronectin and *UDP*-N-acetyl-alpha-D-galactosamine: polypeptide N-acetyl-galactosaminyltransferase 7 (GalNT7) 3′UTRs. Thus, overexpression of nephronectin 3′UTR acted as a miRNA “sponge”, inducing the sequestration of miR-378, which in turn released the inhibition of this miRNA on GalNT7 mRNA [4]. Indeed, few other reports have indicated that GalNT7 is a target for members of miR-30 family and miR-214 and than these miRNAs can modulate the enzyme activity during carcinogenesis [5]–[6].

Here, we have demonstrated that FUT8 can be a target of two miRNAs which are commonly down-regulated during hepatocarcinogenesis [14], [16]. The levels of miR-122 and miR-34a inhibition on FUT8 expression are comparable to that obtained for ALDOA, which is a validated target of miR-122 and which has been widely used to demonstrate the effects of the systemic administration of miR-122 antagonists in the liver [15]. The finding that miR-34a do not modify mRNA levels, while it induces a protein decrease at shorter times after transfection, is not surprising, since it is well known that miRNAs can exert their effects only at a translational level and that these effects can be also reversible [18]. Specifically, miR-34a ectopic expression was shown to affect proteins without perturbing the corresponding mRNA levels in the human HepG2 cell line [16]. This finding underlies that in some cases, microarrays and transcriptomic analyses alone could not reveal the effects mediated by miRNAs.

FUT8 upregulation is not observed in hepatocarcinoma only, but it is overexpressed also in several malignant tumors, including ovarian, thyroid, colorectal and non small cell lung cancer [19]–[22]. Moreover, increased levels have been correlated with progression and severity of the disease [22]. Indeed, core fucosylation has been shown to promote EGFR receptor activation and downstream signalling and, moreover, it can influence the response to tyrosine kinase inhibitors [9]–[11], suggesting a potential relevant role of FUT8 in cancer treatment.

In conclusion, our study highlights the possibility that miRNAs can affect expression levels and contribute to “fine tuning” of the activity of enzymes involved in glycan formation, in particular during development and cancer. This study also point out the need of more detailed studies aimed to understand the impact of non-coding RNA in the control of glycosylation, in particular in cancer. Thus, together with the transcriptional regulation, the contribution of post-transcriptional effects as well should be taken into account in functional glycomic studies.
